# Glances at the Insane in Other Lands.—VIII

**Published:** 1889-04-13

**Authors:** 


### Glances at the Insane in Other Lands.—VIII.

Illinois.—The reports of American asylums very often give elevations and ground plans of the buildings, so that readers not familiar with the institution can form some idea of its construction—a very pleasant and useful prelude to studying it. Much might be learned if the lead of the American asylums in this particular were followed in England and on the Continent.

In the report of the Central Hospital for the Insane for the State of Illinois we find an isometrical perspective of the main building, and an elevation and ground plan of the new building lately annexed to the old one. This annex is for 300 beds, and was built for about £76 a bed, a sum rather lower than the average of large additions hitherto made to our country asylums. We are not now concerned with the plans of asylums, but we may just mention that the annex seems much better arranged than the majority of American asylums, at least so far as we have had an opportunity of examining their plans.

The Central Hospital has been in existence since 1851, and during the thirty-seven or thirty-eight years of its life it has received close on 9,000 patients within its walls. In one of our reviews of American reports we noticed that it was proposed to obtain more room in the hospital by discharging harmless chronic cases to the almshouses or to their homes. The Illinois trustees take a much higher view of their duties, and one much more in harmony with our ideas. They say: "The attempt to care for the insane in almshouses should be abandoned, and adequate provision made for all in the State hospitals. The high modern conception of what is due to humanity demands that all these unfortunates should be placed where they can enjoy the best curative and remedial agencies known to our civilisation." The trustees end their report (an unusually good one, by the way) by saying that the treatment of the insane has occupied the attention of physicians for many years, and that now physicians and scientists should have their studies directed to means for the prevention of insanity. This little bit of advice puts a nice round finish to their report; but the Illinois trustees are quite enlightened enough to know that the advice given by physicians and scientists in such things is hardly ever listened to, much less acted on, by the public. For instance, the statistics of nearly every asylum show that the most potent factors of insanity are hereditary tendency and alcohol. Now alienist physicians are never tired of inveigling against the marriages of those having the insane neurosis, and against the abuse of stimulants; but it would be hard to point to an instance where the warning has had any effect.

The Illinois Hospital is by no means a small one, for it contained 926 patients at the close of 1886, showing an increase of nearly one hundred during the previous biennial period. The admissions were not fewer than 704, and the recoveries reached 141, while 92 died. The deaths, calculated in the usual English method, were only 7 per cent. for 1885 and 5·50 per cent. for 1886. The cost per head per week was 13s. 3d.

Among the improvements spoken of in the report under notice, is the introduction of electric lighting; it being thought not only a better light than gas, but a safer one. We think Dr. Carriel has rather exaggerated the dangers arising from the use of gas. English asylums are almost invariably lighted with it and warmed by open coal fireplaces with no more protection to either than in an ordinary house, and yet it is very rare to hear of a fire originating from either of these sources. The cost of the electric plant is given at twelve thousand dollars.

Dr. Carriel has the following paragraph on the non-restraint system: "The matter of restraints depends largely upon one's faculties for care and the number of attendants employed, and this depends upon the amount of means at the command of the management. With only three dollars and nineteen cents a week per capital a very large force of attendants could not be employed to give up their time to the destructive, or to those that are inclined to denude themselves, or to violence and personal encounters. . . . We strive to find the happy mean in regard to personal liberty, restraints, etc., and trust we are walking therein." This is all very well, and, although we do not object to the use of restraint in surgical cases, we should like to know what is meant by the "happy mean." We do not find any table recording the amount of restraint, so that there may be only one case in a year, or there may be as much as one case in a week. In our notice of the Alabama Hospital, we referred to the total abolition of restraint in that institution, and to the good results which followed the abolition. Of course, no two asylums are exactly alike, either in construction or in the classes of patients admitted, or in the capacity of the attendants employed. Still, it would seem tolerably certain that if restraint can be totally abolished in Alabama it might be reduced to a minimum in Illinois, and we greatly doubt whether its abolition would entail an increase in the staff.

Dr. Carriel takes the hint dropped by the trustees about the desirableness of finding means for preventing insanity, and he gives us some very interesting extracts from the writings of his American and Canadian *confrères*, which we regret want of space forbids quotation in these columns. After naming education as a preventive, and after telling us that the "art of living" should be taught to everyone, with physiology and hygiene, we are informed that it is the foreign-born element in the population from which the great increase of insanity comes. He proves from the census that, whereas only 19 per cent. of the population are foreigners, 41 per cent. of the insanity was among foreigners. This is certainly very alarming, and calls as peremptorily for redress as does the landing of foreign paupers on our own shores. With the States, however, it is but a fringe of great question of "race," which will one day shake the Union to its foundation.

In Illinois the law relating to the care of the insane has some peculiarities worth noting. Before a patient can be admitted to the asylum, a petition must be filed. The judge then issues a writ, whereupon the alleged insane person is brought into court and tried by a jury of six persons, one of whom must be a physician. The alleged lunatic has the right to be heard by counsel, and may challenge jurors. The jury find a verdict and must state the grounds on which they have formed their opinion. If the verdict show that the person is insane an order is made out by the court, and the patient removed to an asylum. There is a special clause to the effect that any superintendent admitting to his asylum, whether pauper or private, any patient not declared insane by a jury, he may be imprisoned for a year or fined five hundred dollars. How this system can work for good in cases of puerperal mania, for instance, it is impossible to see, or how any jury can possibly come to a correct conclusion in some cases of homicidal mania is a mystery to us.

---

In the algide stage of cholera, when the pulse can no longer be felt in the principal arteries, it is possible to secure a return to consciousness, which may be only temporary or may give time for the *vis medicatrix naturæ* to assert herself, by injecting into the veins water of about the same temperature as the blood.

